# Assessment of the capacity of ChatGPT as a self-learning tool in medical pharmacology: a study using MCQs

**DOI:** 10.1186/s12909-023-04832-x

**Published:** 2023-11-13

**Authors:** Woong Choi

**Affiliations:** Department of Pharmacology, College of Medicine, Chungbuk National University, Cheongju, Chungbuk 28644 Korea

**Keywords:** ChatGPT, Large language model, Self-directed learning, Performance, Multiple-choice questions, Rationale, Referencing

## Abstract

**Background:**

ChatGPT is a large language model developed by OpenAI that exhibits a remarkable ability to simulate human speech. This investigation attempts to evaluate the potential of ChatGPT as a standalone self-learning tool, with specific attention on its efficacy in answering multiple-choice questions (MCQs) and providing credible rationale for its responses.

**Methods:**

The study used 78 test items from the Korean Comprehensive Basic Medical Sciences Examination (K-CBMSE) for years 2019 to 2021. 78 test items translated from Korean to English with four lead-in prompts per item resulted in a total of 312 MCQs. The MCQs were submitted to ChatGPT and the responses were analyzed for correctness, consistency, and relevance.

**Results:**

ChatGPT responded with an overall accuracy of 76.0%. Compared to its performance on recall and interpretation questions, the model performed poorly on problem-solving questions. ChatGPT offered correct rationales for 77.8% (182/234) of the responses, with errors primarily arising from faulty information and flawed reasoning. In terms of references, ChatGPT provided incorrect citations for 69.7% (191/274) of the responses. While the veracity of reference paragraphs could not be ascertained, 77.0% (47/61) were deemed pertinent and accurate with respect to the answer key.

**Conclusion:**

The current version of ChatGPT has limitations in accurately answering MCQs and generating correct and relevant rationales, particularly when it comes to referencing. To avoid possible threats such as spreading inaccuracies and decreasing critical thinking skills, ChatGPT should be used with supervision.

**Supplementary Information:**

The online version contains supplementary material available at 10.1186/s12909-023-04832-x.

## Introduction

Created by OpenAI, ChatGPT is an advanced large language model (LLM) that has been pre-trained to chat in natural language [1]. Since its launch in late 2022, ChatGPT has drawn considerable attention from the public. Thanks to its large capacity and training text corpora [2], ChatGPT is able to produce human-like responses, going as far as to demonstrate reasoning through chain-of-thoughts mimicking human problem-solving behavior [3–6]. After ChatGPT met the passing threshold on the United States Medical Licensing Examination (USMLE) [7, 8], many authors applied ChatGPT on answering other multiple-choice questions (MCQs) in the medical domain such as physiology [9], anesthesiology [10, 11], ophthalmology [12], and parasitology [13]. One meta-analysis reported that ChatGPT demonstrated an accuracy of 61.1% (95% CI 56.1%–66.0%) in answering MCQs in medical examinations [14].

Given that MCQs can be used as a self-learning tool [15, 16], such performance suggests that ChatGPT could act as an easy-to-access interactive learning environment, which could lead to greater retention of information and more pleasant learning experience [7].

The Korean Comprehensive Basic Medical Sciences Examination (K-CBMSE) is a minimum competency test taken by Korean medical students who have completed didactic learning and laboratory experiment for basic medical sciences (See Supplement 1 for the details) [17]. One theme of K-CBMSE focuses on pharmacology, which includes MCQs at three levels of cognitive taxonomy: recall, interpretation, and problem-solving [16]. Pharmacology is often perceived as a challenging subject by students due to (1) the introduction of numerous new terms and concepts, and (2) requirement of complex background knowledge such as pathophysiology and biochemistry. Therefore, reinforcement of key concepts by self-learning is essential to improve understanding, learning and retention [18].

ChatGPT was suggested as a self-learning tool for students facing difficulties in learning pharmacology, as it achieved a high accuracy rate when answering centric questions from a pharmacology textbook for undergraduate students [19]. However, ChatGPT’s ability to answer MCQs in pharmacology have not been addressed in the past literature. In this study, the capacity of ChatGPT as a self-learning tool for pharmacology was tested on selected MCQs from the pharmacology section of K-CBMSE. ChatGPT was asked four incrementally designed prompts to provide answers, rationales (reasoning or justification) supporting its answers, references for the rationale, and relevant paragraphs or excerpts from each reference. The accuracy of answers, the soundness of rationales, and the veracity of references and relevant paragraphs were evaluated. Cases of incorrect answers and rationales were identified along with potential causes for the errors. Possible strategies to minimize the drawbacks of ChatGPT were discussed.

## Methods

### Aim

This study assessed ChatGPT’s potential as a standalone self-learning tool for medical pharmacology by evaluating its response to 312 MCQs derived from the K-CBMSE test items. The responses were assessed based on the correctness of answer, rationale, references, and paragraph from each respective reference. As MCQs are a combination of test items and incrementally engineered lead-in prompts, the study also tested whether the cognitive taxonomy level of the test items and the incrementally engineered prompts interacted to influence ChatGPT’s performance.

### Construction of test item dataset

Test items from the K-CBMSE for years 2019 to 2021 (a total of 105 test items) were used as the test item dataset.^1^ Test items with figures (27 items) were excluded because ChatGPT could not interpret images. The remaining 78 test items were translated from Korean to English by the author. During the translation, long Korean sentences were split into short English sentences for better readability, and appropriate plain words or medical terms were used where required. The cognitive taxonomy level of the test items was also rated by the author as recall, interpretation, and problem-solving [16].

### Prompt engineering

For the answer, references for the rationales, relevant paragraphs or excerpts in each reference), lead-in prompts were engineered incrementally for each of the four levels. This four-level prompting is an incremental prompting technique that uses four levels of prompts to guide a large language model (LLM) such as ChatGPT towards a desired response by providing multiple prompts, one after another [20]. It was hypothesized that incremental prompting might increase ChatGPT’s workload and error rate.

1. *Prompt 1 (correct answer): Please choose the best answer for the following question,*
2. *Prompt 2 (rationale): Please choose the best answer for the following question and explain the rationale,*
3. *Prompt 3 (references): Please choose the best answer for the following question and explain the rationale. Please provide the references (Uniform Resource Locator or URL, title, and authors) that support the rationale,*
4. *Prompt 4 (relevant paragraph): Please choose the best answer for the following question and give a rationale for the answer. Please provide the references (URL, title, and authors) that support your rationale. Please provide the relevant paragraphs or formulas from each reference.*

### Multiple-choice question dataset

**Fig. 1:**
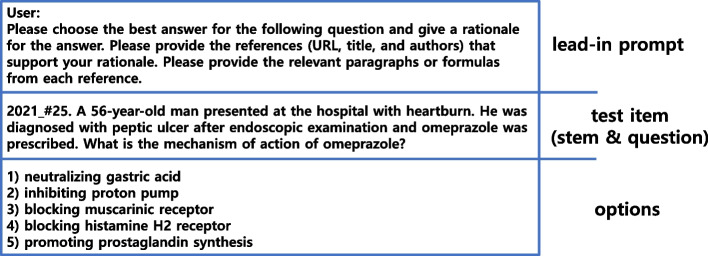
The typical style of each multiple-choice question. Each question consisted of a lead-in prompt, a test item, and options. The lead-in prompt could ask for the answer; a rationale supporting the answer; references; and paragraphs from the references

A single MCQ was composed of one lead-in prompt, one blank line, and the original test item. Figure 1 shows the typical style of each MCQ. Since each test item could be paired with four different lead-in prompts, 78 test items generated a total of 312 MCQs.

### ChatGPT

ChatGPT (versions between Jan/28/2023 and Jan/29/2023; OpenAI) was used. The version of ChatGPT is powered by Generative Pretrained Transformer (GPT)-3.5 and has been pretrained on a large size (from 570 gigabytes to 45 terabytes) of text data [1, 2]. There is no report whether ChatGPT received any additional pharmacology-specific pretraining.

### ChatGPT inquiry and the responses thereof

**Fig. 2:**
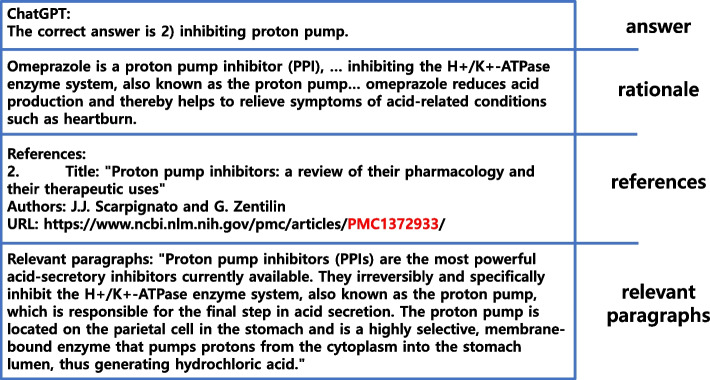
The typical style of ChatGPT’s response. Depending on the level of the lead-in prompt, typical ChatGPT’s response consisted an answer; a rationale supporting the answer; references; and paragraphs from the references

ChatGPT was used to generate responses to English MCQs by feeding one question to the model at a time, deleting previous chat history before each submission to prevent previous questions from influencing the current response. A response consisted of answers, rationales, reference lists, and relevant paragraphs from each reference, depending on the prompt. Figure 2 shows a typical example of ChatGPT’s response style.

### Data summary

ChatGPT-generated responses were evaluated to check whether: (1) the answer for each MCQ was correct; (2) the answers to each test item were consistent over the prompts; (3) ChatGPT returned scientifically accurate and relevant rationales.; (4) reference lists were correct (i.e., ChatGPT provided the accurate citation information for references); and (5) a relevant paragraph could be found in the references listed, if possible. The results were binary (yes or no), and the number of yes and no results was summarized.

### Statistical analysis

All results were expressed in categorical contingency tables and statistical analysis was performed online using Fisher’s exact test (https://astatsa.com/FisherTest/). When cell frequencies were less than 5, the Freeman-Halton extension of Fisher’s exact test was performed using the Free Statistics Calculator v4.0 (https://www.danielsoper.com/statcalc/default.aspx). If required, chi-square goodness-of-fit test was performed using Chi-Square Goodness of Fit Test Calculator (https://stats.libretexts.org/Learning_Objects/02%3A_Interactive_Statistics/36%3A__Chi-Square_Goodness_of_Fit_Test_Calculator). A *p*-value of less than 0.05 was considered statistically significant.

## Results

### Answers

The overall accuracy of ChatGPT’s answers to MCQs was 76.0% (Table 1). Responses with an incorrect answer (65/312, 20.8%), with multiple answers (4/312, 1.3%), or a response of “not determined” (6/312, 1.9%) were grouped as incorrect answers (75/312, 24.0%). Although the accuracies across the prompts varied from 71.8% to 82.1%, they did not differ significantly (See Supplement 2 for the details). ChatGPT’s accuracy was higher than its previously reported performance of 56.1%–66.0% (95% CI) [14] and Korean students’ average performance of 55.3% (See Supplement 1 for the details). In terms of the cognitive taxonomy level of MCQs, the accuracy was 86.4% (152/176) for recall, 77.5% (62/80) for interpretation, and 41.1% (23/56) for problem-solving (See Supplement 2 for the details). Table 1 shows the performance for prompt 4 (relevant paragraph) in each taxonomy level, as the performance for other prompts was not significantly different.

**Table 1:** ChatGPT performance for multiple-choice questions with prompt 4 (relevant paragraph)

Cognitive taxonomy level (Number of test items)	Correct answers to Prompt 4 (relevant paragraph) (%)	Overall accuracy over four lead-in prompts (%)
Recall (44)	81.8	86.4
Interpretation (20)	75.0	77.5
Problem-solving (14)	35.7	41.1
Total test items (78)	71.8	76.0

Please see Table S2-1 in Supplement 2 for the details

To assess the concordance or consistency of answers to test items, the responses were aggregated by test item. The correctness for a single test item were classified as all correct, all incorrect, or partially correct across the prompts, and the all-correct and all-incorrect responses were regarded as concordant responses. Of the 78 test items, 60 items (76.9%) had concordant response across the prompts (Table 2). For partially correct responses, the incorrect-to-correct answer ratio varied between 1-to-3 to 3-to-1 with various correct-incorrect sequences (data not shown).

**Table 2:** The concordance of answers to the test items across prompts

Answers across prompts	The number of test items	%
Concordance		
All correct	49	62.8
All incorrect	11	14.1
Discordance^a^		
Partially correct	18	23.1
Total	78	100.0

^a^Discordant responses are inconsistent or conflicting set of answers to the same test item for repeated inquiries with different prompts

### Rationale

Prompts 2 (rationale) to 4 (relevant paragraph) required the rationale to be included in the response (234 MCQs). The scientific accuracy of each rationale was assessed by the author. Among 234 MCQs, 178 MCQs were correctly answered with either a correct (172/178, 96.6%) or an incorrect rationale (6/178, 3.4%). 56 MCQs were incorrectly answered with either a correct (10/56, 17.9%) or an incorrect rationale (46/56, 82.1%). Overall, 22.2% (52/234) of the rationales were incorrect. Table 3 shows that correct answers were more likely supported by correct rationale and incorrect rationales were more likely to be associated with incorrect answers (*Χ*^*2*^*(df* = *1, N* = *234)* = 152.93, *p* < 0.05).

**Table 3:** Scientific accuracy of rationale for correct and incorrect responses

Answers (N)	Rationale (%)			
	Scientifically accurate	Scientifically inaccurate	Overall	*p*-value
Correct (178)	73.5	2.6	76.1	*p* < 0.05*
Incorrect (56)	4.3	19.6	23.9	
Subtotal (234)	77.8	22.2	100.0	

^*^The Fisher’s exact test and the follow-up chi-squared test (*Χ*^*2*^*(df* = *1, N* = *234)* = 152.93) found significant interaction between the scientific accuracy of rationale and the accuracy of answers to the MCQs (*p* < 0.05)

The incorrect rationales could be grouped into one of the two categories: information errors (28/52, 53.8%) and reasoning errors (24/52, 46.2%). Information errors involved incorrect information or formula in the rationale (See Fig. S3-1 for correct and relevant supporting paragraph; Figs. S3-2 and S3-3 for the errors in the rationale in Supplement 3), while reasoning errors involved failed identification of the cues from the question stem, disregard of the cues in the question stem, or arithmetic errors including unit conversion (See Figs. S3-4 and S3-5 in Supplement 3).

### References

Prompts 3 (references) and 4 (relevant paragraph) required references for the test items (156 MCQs). In total, 274 references were listed (Table 4). The reference lists consisted of URLs including PubMed, articles in journal citation format, and book information. Among these references, 191 (69.7%) had URLs linked to either an irrelevant or a nonexistent site, including PubMed links that did not match the relevant contents. A total of 350 authors were cited, but 59 authors (16.9%) could not be found on PubMed, Amazon, or Google. Even the combination of the existing authors did not find any relevant articles. Although 152 titles of articles or books were given, 148 titles (97.4%) were incorrect. Figure 2 shows a case of errors in referencing. The reference information presented was “Proton pump inhibitors: a review of their pharmacology and their therapeutic uses. Scarpignato, JJ and Zentilin, G. https://www.ncbi.nlm.nih.gov/pmc/articles/PMC1372933/”. The PubMed Central Identifier PMC1372933 was directed to “Preventive Medicine in World War II. Vol. IV. Communicable Diseases Transmitted Chiefly through Respiratory and Alimentary Tracts. Am J Public Health Nations Health. 1959 Jul; 49(7): 969. PMCID: PMC1372933” and none of the listed authors could be found on PubMed, Amazon, or Google. The reference title, ‘Proton pump inhibitors: a review of their pharmacology and their therapeutic uses’, could not be found in a PubMed search. Even after the references were limited to textbooks and the prompts were modified to require actual books, the errors in the information on authors, book chapters, and pages were persistent (data not shown).

**Table 4:** The types and frequencies of reference errors

	Types of errors	N_1_/N_2_ (%)
A	Reference URLs linked to a wrong page or a ‘404 page not available’ (N_1_) among total references listed (N_2_)	191/274 (69.7)
B	The authors not found on PubMed, Amazon (for books), or Google (N_1_) among the authors appearing in the references (N_2_)	59/350 (16.9)
C	Incorrectly titled articles or books (N_1_) among the references (N_2_)	148/152 (97.4)

### Relevant paragraphs

Prompt 4 (relevant paragraph) asked ChatGPT to identify the relevant paragraphs from each reference (78 MCQs). Only 47 MCQs from Table 5 were provided with paragraphs, which are presented in Table 6 (61 paragraphs). The distribution of the paragraph presentation did not differ significantly between correct and incorrect answers (Table 5; *χ*^*2*^*(1, 78)* = 1.35, *p* > 0.05).

**Table 5:** The distribution of paragraphs between the correct and incorrect answers

Answers to the MCQs (N)	MCQs provided with Paragraphs (%)	MCQs not provided with Paragraphs (%)	Overall (%)	*P*-value
Correct (56)	46.2	25.6	71.8	*p* > 0.05*
Incorrect (22)	14.1	14.1	28.2	
Subtotal (78)	60.3	39.7	100.0	

^*^Fisher’s exact test and a follow-up chi-squared test (*χ*^*2*^*(1, 78)* = 1.35) found no significant interaction between the correctness of ChatGPT’s response and whether or not it was provided with relevant paragraphs from the references (*p* > 0.05)

**Table 6:** The credibility of provided paragraphs between the correct and incorrect answers

Answers to the MCQs (N)	Correct and relevant Paragraph %	Correct but irrelevant Paragraph %	Incorrect Paragraph %	Overall %	*P*-value
Correct (49)	75.4	0.0	4.9	80.3	*p* < 0.05*
Incorrect (12)	1.6	9.8	8.2	19.7	
Subtotal (61)	77.0	9.8	13.1	100.0	

^*^The Freeman-Halton extension of Fisher’s exact test found significant interaction between the correctness of ChatGPT’s response and the credibility of paragraphs from the references (*p* < 0.05)

Irrespective of the correctness of the reference, the contents of 61 paragraphs themselves could be grouped into (1) correct and relevant to the answer key (47/61, 77.0%), (2) correct but irrelevant to the answer key (6/61, 9.8%), and (3) incorrect information (8/61, 13.1%). As shown in Table 6, correct and relevant paragraphs are more likely to support correct answers (See the Supplement 3 for the details of each type of paragraph in Table 6; especially Fig. S3-1 for a correct and relevant paragraph, Fig. S3-6 for a correct but irrelevant paragraph, and Fig. S3-7 for an incorrect paragraph).

## Discussion

Based on pharmacology MCQs, this study found that the current version of ChatGPT need to be improved to be used as a standalone self-learning tool. ChatGPT’s overall performance (76%) in this study surpassed the ranges reported in the previous literature (61.1%, 95% CI 56.1%–66.0%) [14]. Its performance may vary depending on the number of subjects covered by each test, the numbers of options per MCQ, as well as the distribution of test items’ cognitive taxonomy. However, its performance below 95% may limit its reliability as a self-learning tool [14]. ChatGPT outperformed Korean students in terms of overall accuracy in its response (76% vs 55%), but performed poorly on problem-solving MCQs (45%) despite its supposed critical thinking and problem-solving abilities. This result suggests that ChatGPT is still limited in its ability to apply critical thinking and reasoning skills to real-world problems.

Another issue with ChatGPT was the randomness of the generated responses. ChatGPT answered 23.1% of the test items inconsistently across the lead-in prompts. While randomness may be useful when generating creative content or exploring different ideas, it can be a critical problem when answering factual questions [21]. A particularly problematic form of randomness is hallucination, a phenomenon where ChatGPT generates plausible-sounding but incorrect or misleading [22–25]. The hallucinations can be caused by training data biases, lack of required information, limited real-world understanding, or algorithmic limitations [26]. The rationales for the answer and the supporting references were especially susceptible to hallucination. Among all the generated rationales, 22.2% were incorrect and involved information errors or reasoning errors. Generated URL links were often incorrect or unavailable (191/274, 69.7%), and some authors could not be found (59/350, 16.9%). Consequently, while ChatGPT did provide paragraphs to some of the paragraph-requiring prompts, it was not possible to evaluate their veracity because most of the reference links were unavailable. This poor performance demonstrates a weakness of ChatGPT as a standalone self-learning tool. In the medical domain, it is crucial to ensure that information is accurate, as errors or inaccuracies can have detrimental consequences [27]. However, any inaccuracies and misinformation in self-study guides cannot be corrected without references, which would lead to erroneous absorption of information that can negatively impact learning outcomes. The absence of appropriate references may also deprive the students of access to additional information, which in turn could limit their comprehension and understanding related to the subject matter [28].

Despite its limitations, ChatGPT could still be useful as a self-study tool when used under supervision [29]. As a part of preparing students for the challenges in the future, they could be trained to critically evaluate and challenge factually incorrect or misleading responses from ChatGPT, such as tracing evidence to its primary sources to verify the model’s assertions [28]. For instance, students can ask ChatGPT for its chain of thoughts through prompts such as “Explain your reasoning process for the answer” or “Explain your chain of thoughts for the answer” [30]. The responses to these prompts can help students understand ChatGPT’s reasoning, think critically about the underlying information, and develop their own reasoning and critical thinking skills based on the experience. ChatGPT can be an engaging way of learning, but it is important to use it in moderation and not let it replace independent thinking. Students should be cautioned against overreliance on ChatGPT, as it could impair their higher-order cognitive skills, such as creativity and problem-solving [31].

This study contributed to the previous literature by providing evidence that the current version of ChatGPT is not suitable as a standalone self-learning tool and exploring the potential for supervised use of ChatGPT.

However, this study also has several limitations. Firstly, the study employed only 78 test items derived from the K-CBMSE pharmacology. While the sample size is adequate for the purposes of this study, it is still relatively small and may not fully represent all categories of medical examination questions. As a result, future research may seek to utilize a larger and more diverse set of medical examination questions for a more comprehensive evaluation of ChatGPT’s capabilities. Secondly, this study’s primary focus was centered on examining ChatGPT’s capacity to address medical examination MCQs, specifically those pertaining to pharmacology. The outcomes of this research may not necessarily be generalizable to other types of inquiries or domains. To enhance the transferability of the study’s results, subsequent investigations may explore ChatGPT’s efficacy in answering questions in fields other than medicine or in other answer formats such as essays. This approach would aid in establishing the generalizability of the findings and providing more robust support for future practical applications. Thirdly, ChatGPT is rapidly evolving. Significant advancements have occurred during the research process, which could potentially make some findings less relevant. For example, GPT-4 was released while this research was underway, and it is known to be significantly more powerful than ChatGPT [32]. Fourthly, overall performance of ChatGPT may have been overestimated in this study due to the imbalanced distribution of cognitive taxonomy levels in the test items. Only 17.9% of the test items are problem-solving, while 56.4% are recall. To ensure fair comparison across studies, the distribution of cognitive taxonomy levels should be standardized. Finally, there are several key components that can contribute to the effectiveness of learning tools, such as students’ perception and interaction [33, 34]. This study did not assess ChatGPT’s efficacy on these dimensions.

The introduction of new technologies such as internet, mobile devices, and ChatGPT in education presents both opportunities and threats. The introduction of new technologies such as internet, mobile devices, and ChatGPT presents both opportunities and threats in education. Artificial intelligence (AI) technology has the potential to revolutionize education [35], offering personalized virtual assistants and adaptive learning experiences for every student [31, 36, 37]. AI-powered systems can provide timely and immediate feedback, tailored recommendations, and interactive and engaging learning activities [26, 36, 38]. Although some may fear the threats of plagiarism and misinformation posed by ChatGPT, efforts to ban emerging technologies in higher education have been futile historically. ChatGPT is unlikely to be an exception [39].

Instead, we should embrace ChatGPT and other language models as self-learning tools while striving to minimize the associated risks. One possible approach is to develop strategies for appropriate supervision. For example, students can ask ChatGPT to generate a solution to a complex problem, and then evaluate the solution to determine its feasibility or effectiveness [30]. Such setups would require the students to use their problem-solving skills and to think critically about the different factors involved in the problem. By doing so, we can stimulate students’ learning and motivate them to develop higher cognitive skills such as critical thinking and problem-solving. Empirical studies also should be performed to investigate whether using ChatGPT with supervision can truly improve critical thinking and problem-solving skills.

## Conclusion

The current version of ChatGPT has limitations as a useful self-study tool despite its performance in correctly answering MCQs. The answers could be inconsistent when the same inquiry is repeated; the generated rationale could be incorrect; and the generated references were nonsensical. To maximize the potential benefits of AI technology while minimizing its risks, it is imperative to develop effective supervision and moderation strategies.

### Supplementary Information

**Additional file 1: Supplement 1.** Korean Comprehensive Basic Medical Sciences Examination (K-CBMSE). **Supplement 2.** ChatGPT’s accuracy across the prompts. **Supplement 3.** The cases of incorrect responses.

## Data Availability

The datasets used and/or analyzed during the current study are available from the corresponding author on reasonable request.

## Footnotes

1 The test items of K-CBMSE are not publicly available, but can be provided by the Medical Education Assessment Corporation (bmec.ac.kr) on demand for educational or research purposes.
