# Supplementary material for: Assessment of the capacity of ChatGPT as a self-learning tool in medical pharmacology: a study using MCQs
Source: BMC Med Educ. 2023 Nov 13;23:864. doi: 10.1186/s12909-023-04832-x (PMC10644619; doi:10.1186/s12909-023-04832-x)

**Supplement 1. Korean Comprehensive Basic Medical Sciences Examination (K-CBMSE)**

To conform to previous literature [S1], the Korean Basic Medical Science Comprehensive Assessment (K-BMSCA) was renamed the Korean Comprehensive Basic Medical Sciences Examination (K-CBMSE).

K-CBMSE is a comprehensive assessment of basic medical science knowledge for medical students who have completed the prerequisite curriculum. It covers Anatomy, Biochemistry, Microbiology, Parasitology, Pathology, Pharmacology, and Physiology. The test measures whether students have acquired the minimum competencies required for medical practice [1].

K-CBMSE has been developed by the Medical Education Assessment Corporation (MEAC) in Korea. MEAC has kindly granted permission for K-CBMSE test items to be used for research and for some of its test items to be disclosed in this research manuscript. A test item in the K-CBMSE consists of one stem and five options with the task of choosing the best option. MEAC kindly provided the difficulty index and discrimination index for each test item, which are used for controlling the quality of its test items.

Difficulty index or item difficulty is a measure of how difficult a test item is for a given group of test takers. It is calculated as the percentage of test takers who answer the item correctly. Difficulty index is also known as passing proportion or percentage (*P*). For any given group of test takers, the average score on a test is equal to the average difficulty of its items [2].

The discrimination index is a measure of how well an item can discriminate between high- and low-performing test-takers. It is calculated by comparing the performance of students on a particular item to their overall performance on the test. Items with high discrimination indices are those that are more likely to be answered correctly by high-performing students and incorrectly by low-performing students. Point-biserial correlation coefficient (*r_pb_*) is the most common measure of item discrimination. The point-biserial correlation coefficient is mathematically equivalent to the Pearson correlation coefficient when item scores are dichotomous (e.g., pass/fail) and the criterion measure is continuous (e.g., total test score) [3]. It is used in the analysis of item discrimination as the discrimination index (D) [2].

Interpretation of Difficulty Index and Discrimination Index [4]:

Difficulty index (*P*) or facility index if

*P* < 20% Difficult

20% ≤ *P* < 75% Acceptable

*P* ≥ 75% Easy

Discrimination index (D) if

D = Negative. Defective item/wrong key

D < 0.2 Poor discrimination

0.2 ≤ D < 0.4 Acceptable/Good discrimination

D ≥ 0.4 Excellent discrimination.

Figure 1 shows that 14.1% of the 78 test items were easy (*P* ≥ 75%), 1.3% were difficult (*P* < 20%), and 84.6% were within an acceptable range (20-75%). Figure 2 shows that 26.9% of the test items had poor discrimination power (D < 0.2), 2.6% had excellent discrimination (D ≥ 0.4), and 69.2% had acceptable or good discrimination (0.2-0.4). The maximum discrimination (D = 0.4) was observed for items in the acceptable range (20% ≤ *P* < 75%).

**Figure S1-1. Difficulty index of each test item**


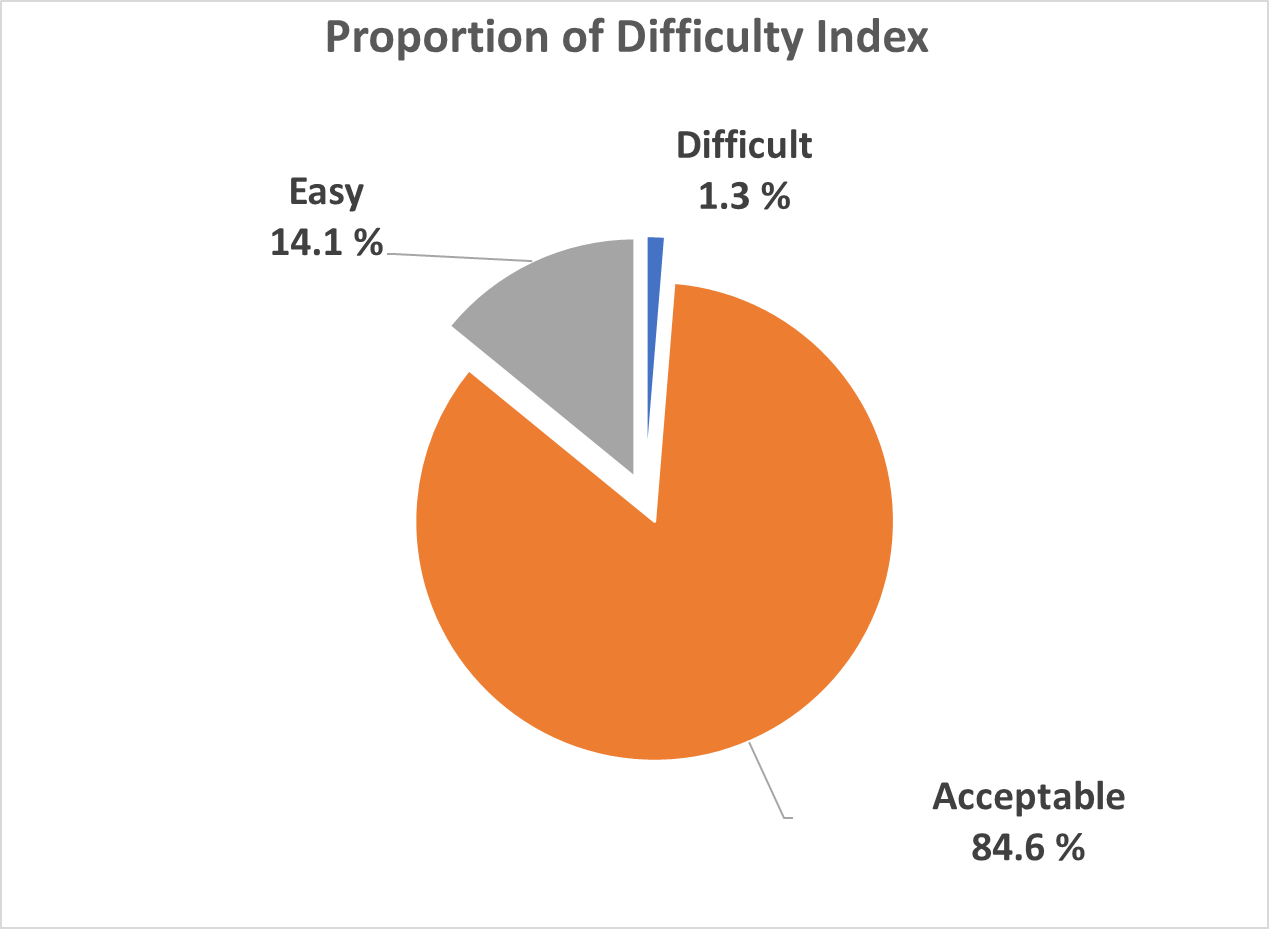


**Figure S1-2. Discrimination index of each test item**


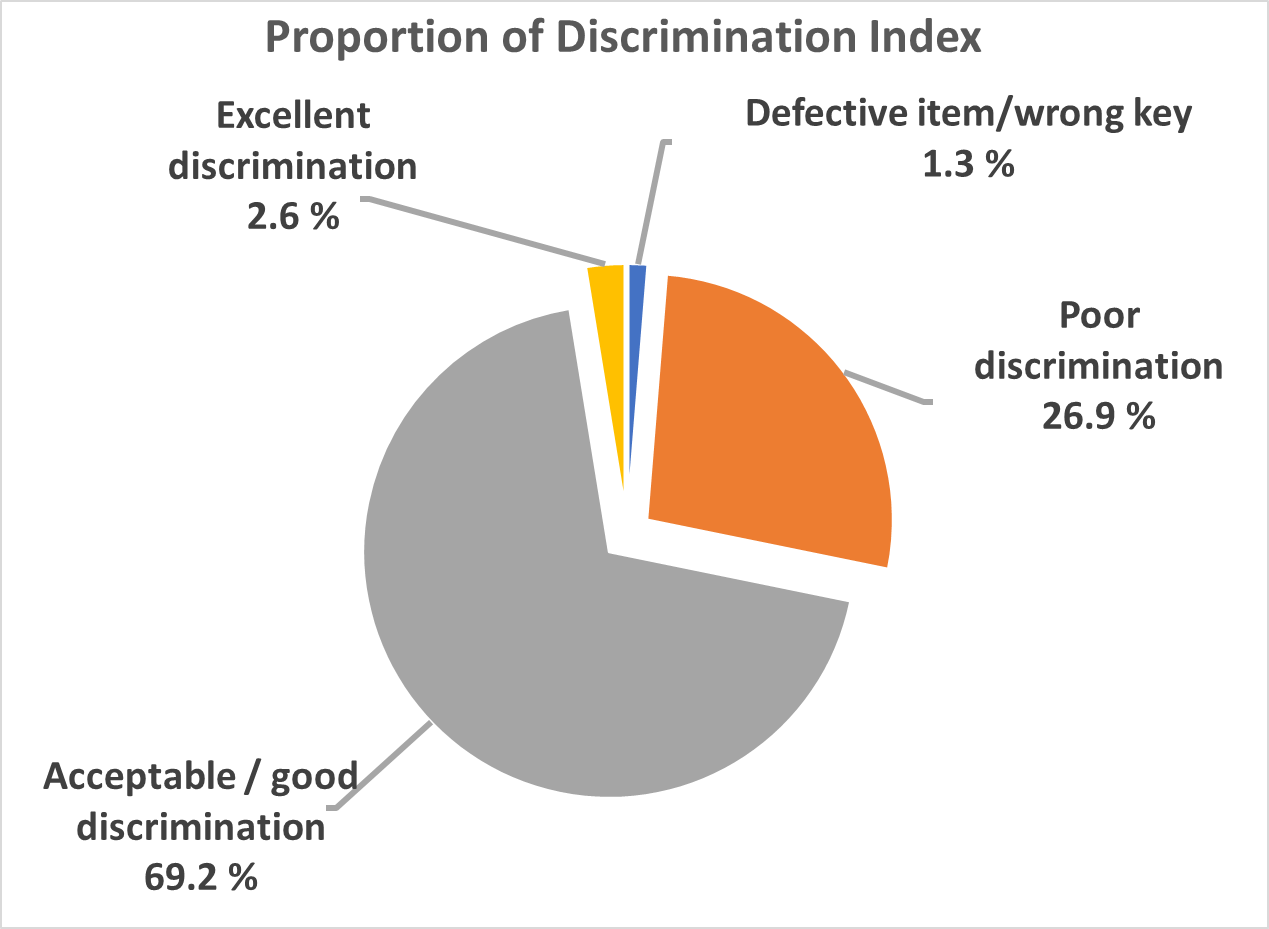


**Table S1-1. Difficulty index across the cognitive taxonomy levels**

| cognitive taxonomy levels  (the number of test items) | The difficulty index for  Korean students | | | ChatGPT | |
| --- | --- | --- | --- | --- | --- |
|  | mean | Standard deviation | 95% confidence interval | Accuracy of  prompt 4 (relevant paragraph) | percentile compared to Korean students |
| Recall  (N, 44) | 0.61 | 0.18 | 0.5568, 0.6632 | 0.82 | 88.2 |
| Interpretation  (N, 20) | 0.49 | 0.20 | 0.4023, 0.5777 | 0.75 | 90.1 |
| Problem-solving  (N, 14) | 0.45 | 0.15 | 0.3714, 0.5286 | 0.36 | 25.5 |
| Overall  (N, 78) | 0.55 | 0.19 | 0.5078, 0.5922 | 0.72 | 81.1 |

Test items are classified by cognitive taxonomy level: recall (44/78, 56.4%), interpretation (20/78, 25.6%), and problem-solving (14/78, 17.9%). The overall performance of Korean students was 55% (95% confidence interval: 50.78%, 59.22%).

ChatGPT performed better than Korean students on recall and interpretation MCQs with prompt 4 (relevant paragraph), with percentile scores of 88.2% and 90.1%, respectively. However, its performance on problem-solving MCQs with prompt 4 (relevant paragraph) was significantly lower at only 25.5%, resulting in an overall percentile score of 81.1% (Table 1).

Cronbach’s alpha has been used as a reliability index by K-CBMSE [5]. Cronbach's alpha for the original test items in 2019, 2020, and 2021 was 0.728, 0.631, and 0.761, respectively. The Medical Education Assessment Corporation in Korea (MEAC) provided informaticians for difficulty and discrimination indices only, so Cronbach's alpha could not be calculated separately for the 78 test items used in this study.

**References**

1. Lee YM, So YH, Ahn DS, Rhee KJ, Im H. Psychometric Analysis of Comprehensive Basic Medical Sciences Examination. Korean J Med Educ. 2002;14(2):301-306. doi: https://doi.org/10.3946/kjme.2002.14.2.301 (in Korean).
2. Urbina S. Chapter 6. In: Essentials of Psychological Testing (Essentials of Behavioral Science). 2nd ed. Hoboken, NJ: John Wiley & Sons, Inc.; 2014.
3. Point-biserial correlation coefficient. [Internet]. Wikipedia. Retrieved from https://en.wikipedia.org/wiki/Correlation_coefficient. Accessed 2023 Aug 30.
4. Rao C, Kishan Prasad HL, Sajitha K, Permi H, Shetty J. Item analysis of multiple-choice questions: Assessing an assessment tool in medical students. Int J Educ Psychol Res. 2016;2(4):201-4.
5. Tavakol M, Dennick R. Making sense of Cronbach's alpha. Int J Med Educ. 2011;2(1):53-55. doi: 10.5116/ijme.4dfb.8dfd.

**Supplement 2. ChatGPT’s accuracy across the prompts**

When the correct answers to MCQs were segmented based on the lead-in prompts, there was no significant interaction between the prompt level and the cognitive taxonomy level (*χ*2(6, 237) = 0.11, *p* > 0.05), which implies that the level of the lead-in prompt did not significantly affect ChatGPT's performance on MCQs of different cognitive levels. However, the chi-square goodness-of-fit test showed significant differences in the cognitive taxonomy of MCQs (*χ*2(2, 237) = 75.33, *p* < 0.001) and no significant differences in prompt level (*χ*2*(3, 237)* = 0.59, *p* = 0.90). ChatGPT performed better on recall (86.4%) and interpretation MCQs (77.5%) than on problem-solving MCQs (41.1%). It is likely that ChatGPT's performance on MCQs is dependent on the cognitive level of the MCQs. ChatGPT likely performed poorly on MCQs that required multiple steps of thought or a chain of thoughts, such as problem-solving type MCQs.

**Table S2-1. The accuracy of ChatGPT’s answers across the cognitive taxonomy levels and prompt levels**

| The cognitive taxonomy level of test items | Prompt 1  (correct answer):  N  (%) | Prompt 2  (rationale)  N  (%) | Prompt 3  (references)  N  (%) | Prompt 4  (relevant paragraph)  N  (%) | Overall accuracy  (%) | *p*-value |
| --- | --- | --- | --- | --- | --- | --- |
| Recall  (N, 44) | 38  (86.4) | 37  (84.1) | 41  (93.2) | 36  (81.8) | (86.4)*** | *p* > 0.05* |
| Interpretation  (N, 20) | 15  (75.0) | 15  (75.0) | 17  (85.0) | 15  (75.0) | (77.5)*** |  |
| Problem-solving  (N, 14) | 6  (42.9) | 6  (42.9) | 6  (42.9) | 5  (35.7) | (41.1)*** |  |
| Accuracy by each prompt  (N, 78) | 59**  (75.6) | 58**  (74.4) | 64**  (82.1) | 56***  (71.8) | (76.0) |  |
| *: The Fisher’s exact test and the follow-up chi-squared test (*χ^2^(6, 237)* = 0.11, *p* = 1.00) did not find any significant interaction between the level of the prompts and the cognitive taxonomy level of test items (*p* > 0.05).  **: The chi-square goodness-of-fit test showed no significant differences in prompt level (*χ^2^(3, 237)* = 0.59, *p = 0.90*)  ***: The chi-square goodness-of-fit test showed significant differences in the cognitive taxonomy of MCQs (*χ*2(2, 237) = 75.33, *p* < 0.001). | | | | | | |

**Supplement 3. The cases of incorrect responses**

In Figure S3-1, the answer and rationale were scientifically accurate, but the supporting paragraph did not cite a valid reference. The rationale and paragraph from the reference described well the mechanism of action of allopurinol (the answer key), but the reference information was directed to a non-existing article. The intended reference was likely “An Update on Safety and Side Effects of Cannabidiol: A Review of Clinical Data and Relevant Animal Studies”, which was published in Cannabis and Cannabinoid Research in 2017 (PMC5569602).

**Figure S3-1. Correct answer with correct and relevant supporting paragraph from the reference.**


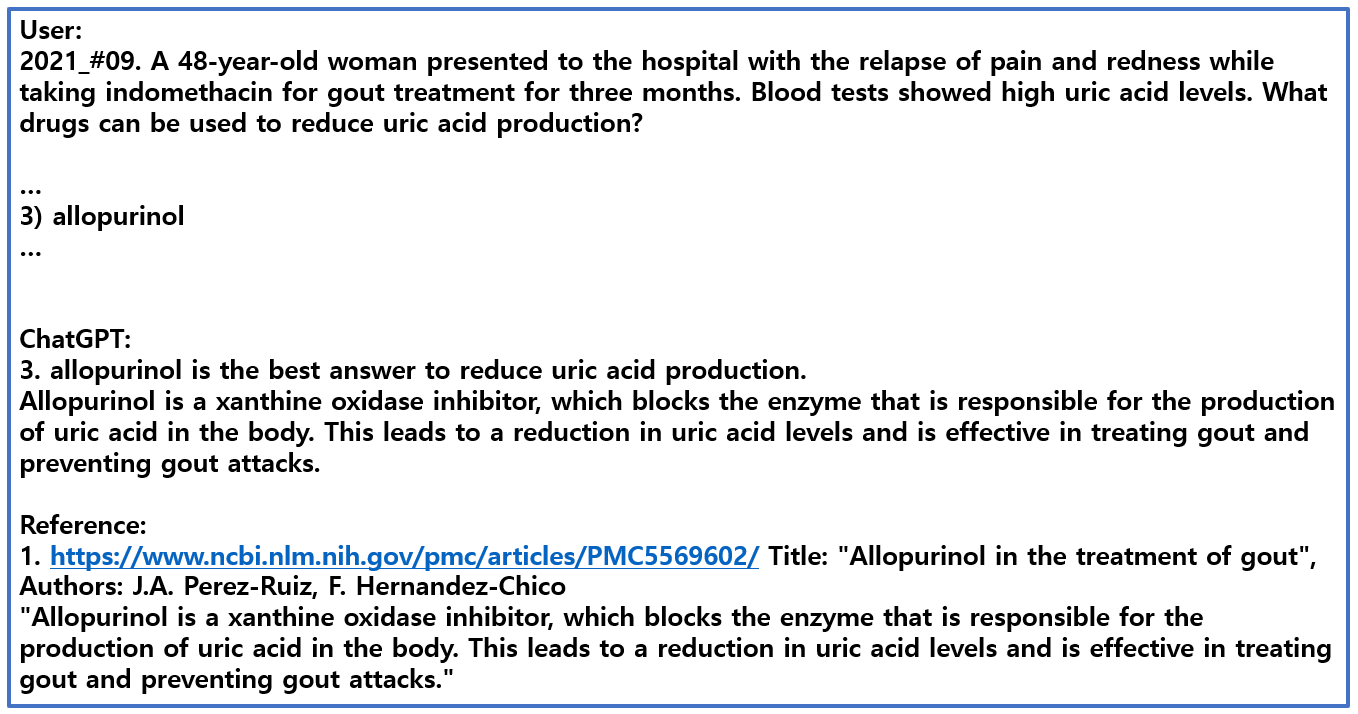


In Figure S3-2, ChatGPT incorrectly claims that propofol is a mu-opioid receptor antagonist. Propofol is a general anesthetic that primarily acts by potentiating the effects of GABA_A receptors. It may also have some minor effects on other neurotransmitter systems, including the opioid system.

**Figure S3-2. Incorrect answer with incorrect information in the rationale**

The answer key is option 1. The errors in the rationale were highlighted in red.


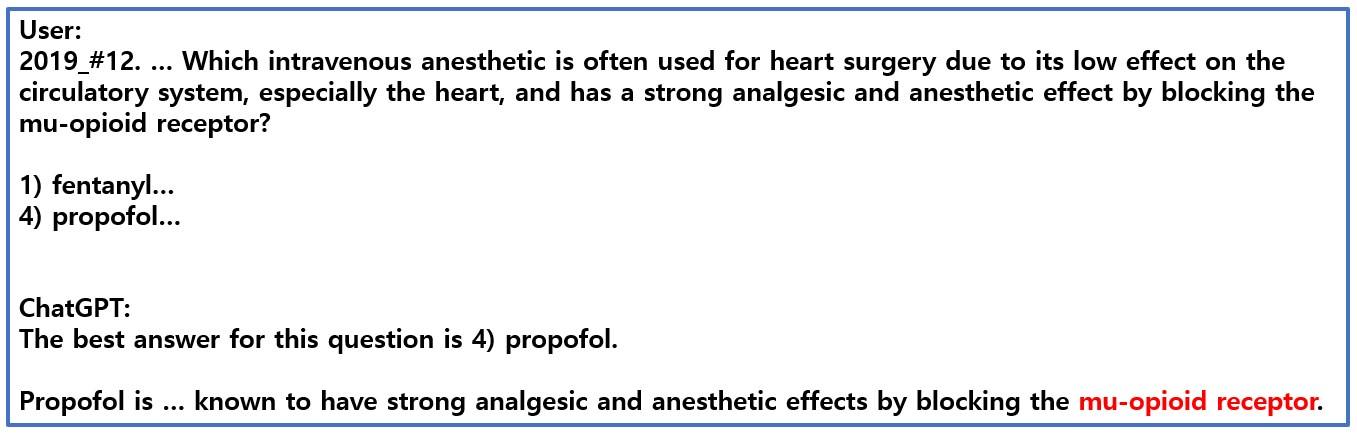


The volume of distribution is the apparent volume of fluid in which the drug is distributed throughout the body. Bioavailability is a measure of how much of the drug reaches the bloodstream after it is administered. According to Figure S3-3, ChatGPT's justification for the loading dose formula is flawed. Clearance, which is the rate at which the drug is removed from the body, should be replaced with bioavailability. The correct formula for the loading dose is therefore:

Loading dose = Target concentration * Volume of distribution / Bioavailability

**Figure S3-3. Incorrect answer with a flawed formula in the rationale**

The error in the formula was highlighted in red.


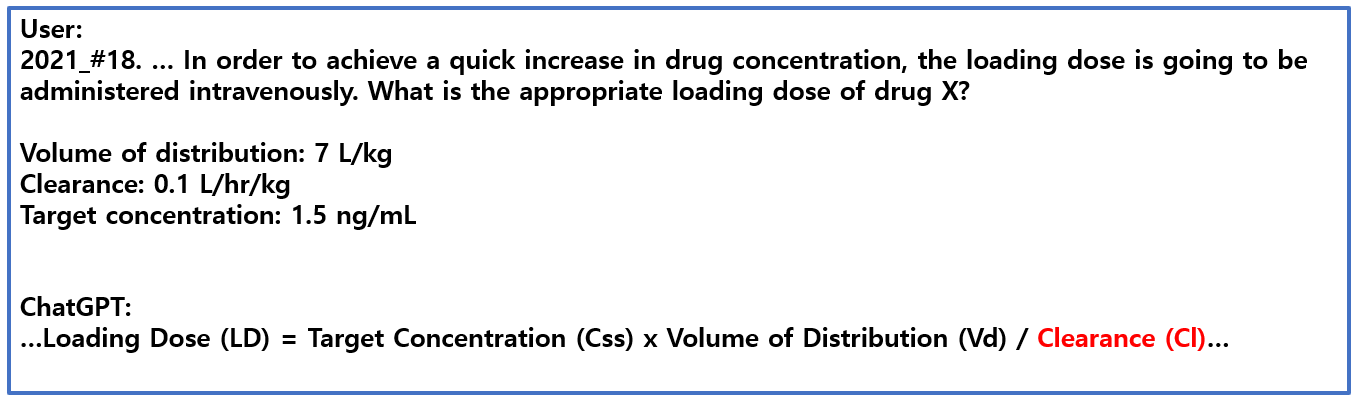


ChatGPT sometimes missed clues in the question stem. In Figure S3-4, ChatGPT likely failed to recognize that the patient had hyperammonemia. In this case, osmotic laxatives such as lactulose can be a treatment option. Lactulose is a synthetic disaccharide that is not absorbed by the small intestine. It is broken down by colonic bacteria into organic acids, which lower the pH of the colon and promote the growth of acid-producing bacteria. This acidic environment inhibits the growth of ammonia-producing bacteria and promotes the trapping of ammonia in the colon. Lactulose also has an osmotic effect, which draws water into the colon and increases stool frequency. This helps to remove ammonia from the body in the feces.

**Figure S3-4. Incorrect answer with reasoning errors in the rationale**

The answer key is option 1. The missed cue and errors in the answer were highlighted in red.


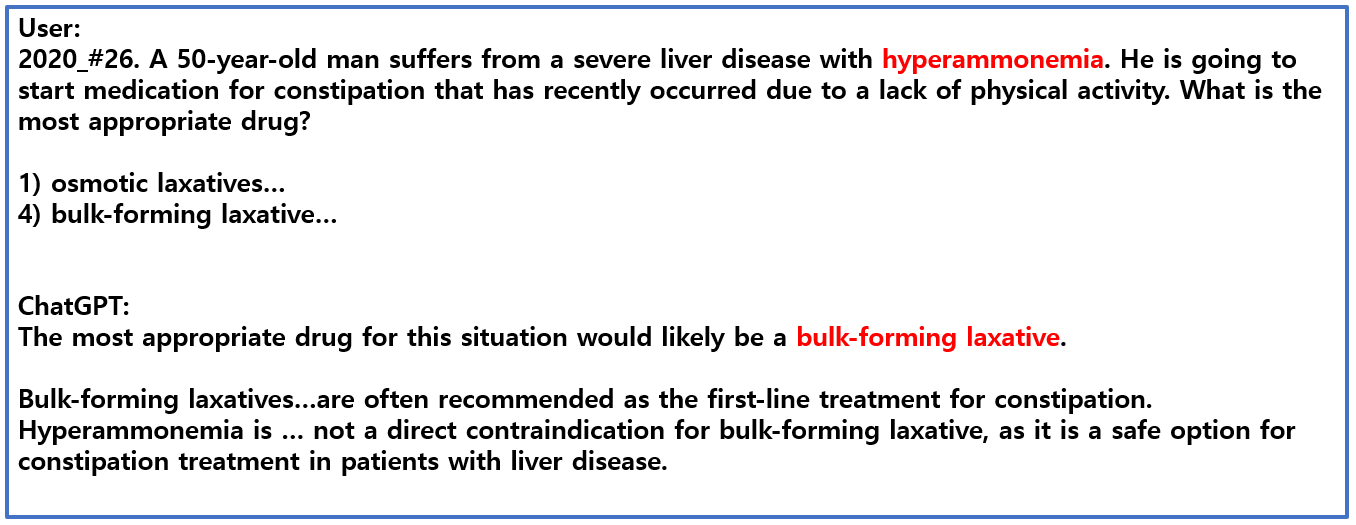


In Figure S3-5, ChatGPT made a unit conversion error when it stated that 10 mg/L * 10 L/kg = 100 mg/L.. Instead, this should be 100 mg/kg. ChatGPT made another unit conversion error when It concluded that the loading dose should be 7000 mg/kg, but had stated the loading dose to be 7000 mg in the previous line.

**Figure S3-5. Incorrect answer with reasoning errors in unit conversion.**

The answer key is option 2. The unit conversion errors were highlighted in red.


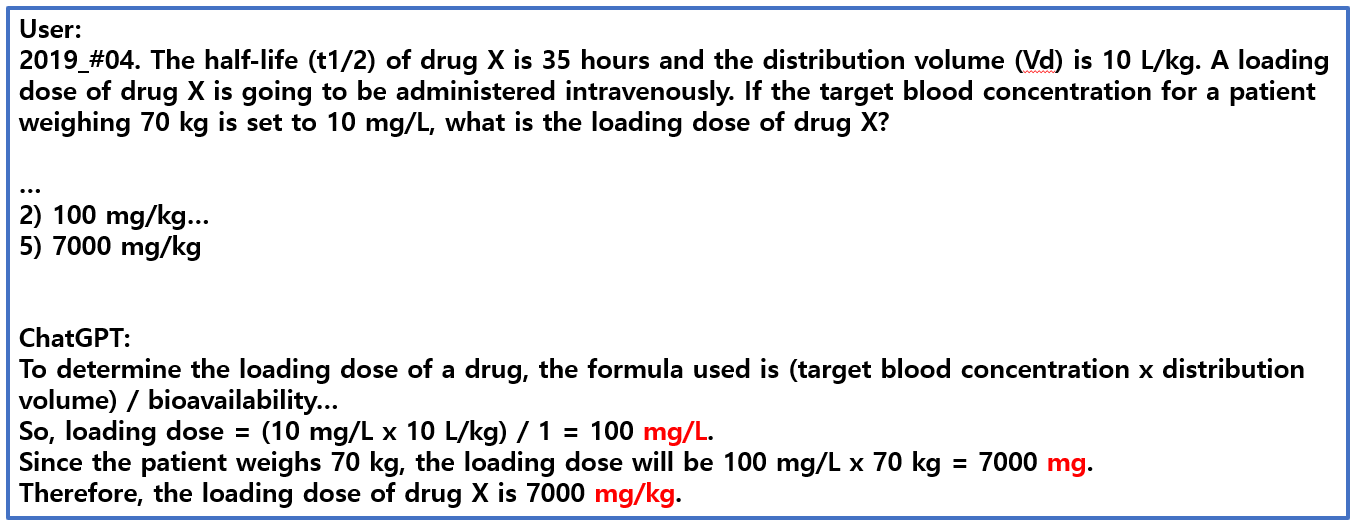


Good Clinical Practice (GCP) requires researchers to respect the subject's right to withdraw from participation in a clinical trial at any time, without penalty or coercion. Option 2 in Figure S3-6 advertises the amount of monetary reimbursement, which is a violation of GCP. Therefore, option 1 is the answer key and option 2 is an incorrect answer to the question. The ChatGPT-provided rationale is in line with GCP and irrelevant to the answer key.

**Figure S3-6. Incorrect answer with correct but irrelevant rationale and paragraph to the answer key.**

The answer key is option 1. ChatGPT likely neglected that advertising the amount of monetary reimbursement (shown in red) could be a violation of GCP. The correct but irrelevant paragraph and rationale was highlighted in red.


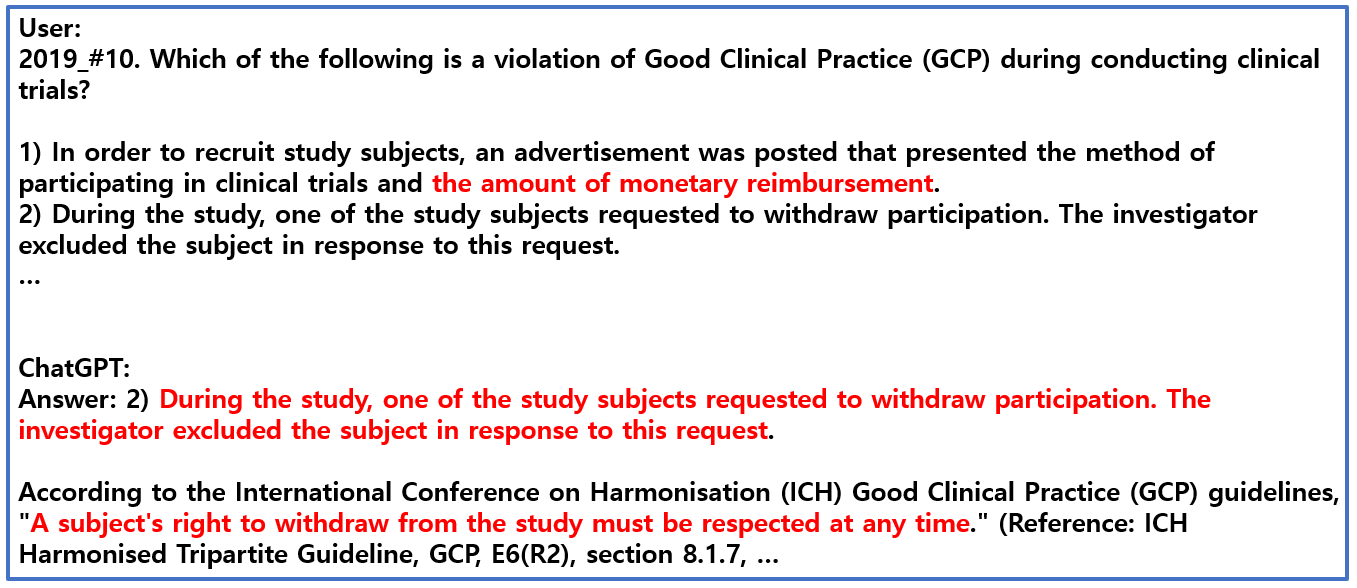


According to the Centers for Disease Control and Prevention (CDC), isoniazid (option 2; answer key) is the only drug recommended for 6-month or 9-month treatment of latent tuberculosis. Rifampin plus isoniazid are only recommended for 3-month treatment. Rifampin only is recommended as a 4-month therapy. In Figure S3-7, ChatGPT likely neglected the therapy duration (9-month) in the question stem. ChatGPT's answer was incorrect, and the paragraph from the reference was not supported by textbook evidences.

**Figure S3-7. Incorrect answer with incorrect paragraph from the reference**

The answer key is option 2. The inaccurate paragraph from the reference is highlighted in red.


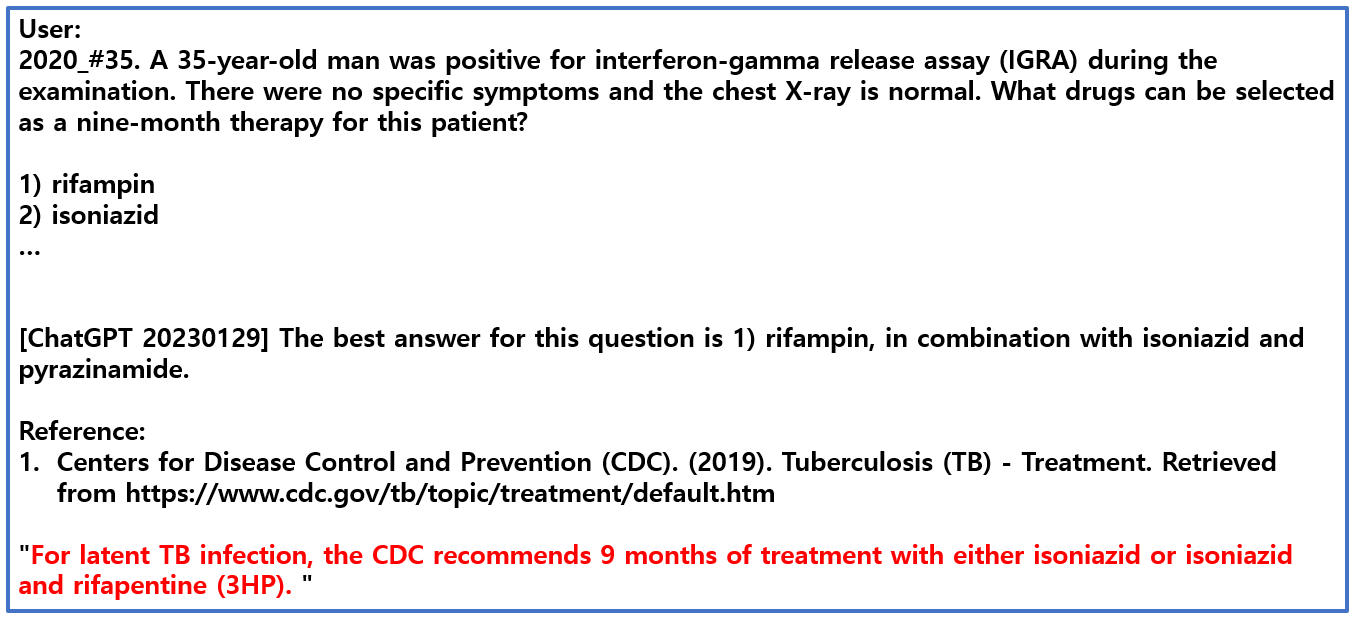

Supplement: Supplementary file 1 — Additional file 1: Supplement 1. Korean Comprehensive Basic Medical Sciences Examination (K-CBMSE). Supplement 2. ChatGPT’s accuracy across the prompts. Supplement 3. The cases of incorrect responses. [file 12909_2023_4832_MOESM1_ESM.docx]
